# Sterile inflammation induced by Carbopol elicits robust adaptive immune responses in the absence of pathogen-associated molecular patterns

**DOI:** 10.1016/j.vaccine.2016.03.025

**Published:** 2016-04-27

**Authors:** Kate H. Gartlan, George Krashias, Frank Wegmann, William R. Hillson, Erin M. Scherer, Philip D. Greenberg, Stephanie C. Eisenbarth, Amin E. Moghaddam, Quentin J. Sattentau

**Affiliations:** a Sir William Dunn School of Pathology, University of Oxford, South Parks Road, Oxford OX1 3RE, UK; b Fred Hutchinson Cancer Research Center, Seattle, WA 98109, USA; c Department of Immunobiology, Yale University, New Haven, CT 06519-1612, USA

**Keywords:** Vaccine adjuvant, Polyanionic carbomer, Th1 immune responses, Antibodies, HIV-1

## Abstract

Carbopol is a polyanionic carbomer used in man for topical application and drug delivery purposes. However parenteral administration of Carbopol in animal models results in systemic adjuvant activity including strong pro-inflammatory type-1 T-cell (Th1) polarization. Here we investigated potential pathways of immune activation by Carbopol by comparison with other well-characterized adjuvants. Carbopol administration triggered rapid and robust leukocyte recruitment, pro-inflammatory cytokine secretion and antigen capture largely by inflammatory monocytes. The induction of antigen specific Th1 cells by Carbopol was found to occur *via* a non-canonical pathway, independent of MyD88/TRIF signaling and in the absence of pattern-recognition-receptor (PRR) activation typically associated with Th1/Ig2a induction. Using multispectral fluorescence imaging (Imagestream) and electron microscopy we demonstrated that phagocytic uptake of Carbopol particles followed by entry into the phagosomal/lysosomal pathway elicited conformational changes to the polymer and reactive oxygen species (ROS) production. We therefore conclude that Carbopol may mediate its adjuvant activity *via* novel mechanisms of antigen presenting cell activation and Th1 induction, leading to enhanced IgG2a responses independent of microbial pattern recognition.

## Highlights

• Carbopol induces Th1/IgG2a responses without PRR activation.
• Carbopol polymer morphology is changed by APC phagocytosis leading to ROS induction.
• This study highlights a potentially novel mechanism for *in vivo* cellular activation.

## 1 Introduction

Adjuvants are essential components of vaccines in which the vaccine antigen lacks robust intrinsic immunogenicity, such as those composed of recombinant or purified subunits of pathogens, or tumor antigens that may require tolerance to be broken. In recent years the adjuvant field has made breakthroughs in understanding underlying mechanisms of adjuvant activity, with the discovery of multiple pattern recognition pathways triggering innate and adaptive immune activation [1], [2]. This has led to the discovery of numerous molecules with adjuvant activity that stimulate the immune system *via* defined pathways, including those triggered by toll-like receptors (TLRs) [1], [2], the NLRP3 inflammasome [3], [4] and IRF3 [5], [6]. Some of these molecules have made their way into clinical trials and have considerable promise in vaccine development. Surprisingly, however, some of the most well-known and longstanding adjuvants, including aluminum salts ‘alum’, oil-in-water emulsions such as Freund's adjuvants, and MF59 appear to act by mechanisms at least partially distinct from these pathways [7], [8], [9]. Other potential modes of adjuvant action are hypothesized to include less specific activities such as the ‘depot’ effect by which the adjuvant sequesters antigen and releases it into the system over time, and local tissue damage resulting in release of intracellular inflammatory mediators such as ATP, nucleic acids, uric acid, IL-25 and IL-33 [6], [10].

The immune-modulating activities of polyanions were first described over 30 years ago [11], [12] and more recently, polyacrylic acid polymers termed carbomers have been evaluated as adjuvants in veterinary vaccines [13], [14], [15], [16], [17], [18]. These reports suggest that carbomers are not harmful in mammals and are more effective than antigen alone. Carbopols have been combined with other adjuvant formulations such as MF59 to yield additive or potentially synergistic adaptive immune responses [19], [20], and Carbopol is a component of the commercially-available adjuvant Adjuplex™ (Advanced BioAdjuvants) [21] and a licensed veterinary vaccine in pigs (Suvaxyn, Wyeth). We have previously demonstrated that Carbopol elicits strong Th1-type T and B-cell responses in mice, mediating protection from otherwise lethal influenza infection, and anti-tumor responses [22]. We observed that Carbopol did not have obvious toxicity in mice [22] or non-human primates [23], and propose that this type of polymer might have utility as a human vaccine adjuvant.

Here, we establish mechanistic insight into Carbopol's adjuvant effects, identifying strong inflammatory responses, cellular recruitment and phagocyte uptake of Carbopol, and identify phagocytosis as a key checkpoint in the immune response to Carbopol, resulting in changes to the physical properties of the adjuvant and disruption of the lysosomal pathway. We conclude that Carbopol utilizes a novel mechanism of APC activation *in vivo* resulting in potent adaptive immune responses to co-administered antigen.

## 2 Materials and methods

### 2.1 Antigens, adjuvants and immunization

HIV-1 envelope glycoprotein (Env)-based recombinant soluble gp140 (<0.05 EU/mL endotoxin) was derived from HIV-1_97CN54_ (Polymun Scientific Inc.). Pre-conjugated ovalbumin (OVA)-AF647 (Molecular Probes) was reconstituted in endotoxin-free PBS (Gibco) prior to use. A 2% (w/v) Carbopol-974p stock (Particle Sciences Inc., UK) was prepared from powder in endotoxin-free PBS, neutralized to pH 7.2 with NaOH. Carbopol preparations contained <0.05 EU/mL of endotoxin, assayed by Lonza Cologne GmbH. Alhydrogel adjuvant (Brenntag Biosector) was diluted in endotoxin-free PBS prior to injection. Balb/c, 129S6/SvEv and 129S6/SvEv.MyD88^−/−^ mice were bred at the University of Oxford. C57BL/6, C57BL/6.NLRP3^−/−^ and C57BL/6.Caspase1^−/−^ mice were bred at Yale University. C57BL/6, C57BL/6.TRIF^−/−^ and C57BL/6.MyD88^−/−^TRIF^−/−^ mice were bred at The Fred Hutchinson Cancer Research Center. All mice used in this study were age and sex-matched within each experiment and procedures were performed under the appropriate licenses in accordance with the UK Animals (Scientific Procedures) Act 1986 with local ethical approval.

### 2.2 Leukocyte phenotyping

Peritoneal leukocytes were isolated by sequential small (2 mL) and large (5 mL) volume peritoneal lavages and supernatant from small volume lavages used in cytokine/chemokine analyses. Cell fractions from both lavages were pooled and analyzed by flow cytometry. The absolute numbers of B-cells (CD11b^−/int^CD19^+^), T-cells (CD11b^−^CD3^+^), monocytes (CD11b^hi^Ly6C^++^Ly6G^−^F4/80^int^), macrophages (CD11b^+^F4/80^hi^Ly6G^−^Ly6C^−^), neutrophils (CD11b^+^Ly6G^hi^Ly6C^+^F4/80^−^), eosinophils (CD11b^+^Ly6C^lo^Ly6G^int^F4/80^lo^SSC^hi^), and dendritic cells (DC) (CD11b^−/int^CD11c^+^F4/80^−/lo^MHC-II^hi^) were determined. To reduce non-specific antibody binding, cells were pre-incubated in Mouse Fc-Block (BD Biosciences). Flow cytometry was performed using either FACSCalibur (BD Biosciences) or CyAN ADP cytometers (Beckmann Coulter, USA) and data analyzed *via* FloJo software (TreeStar Inc., USA).

### 2.3 Antibody/cytokine/chemokine detection

Serological analyses for antigen-specific antibodies were performed as previously described [22]. Supernatants were separated from either peritoneal lavage or cultured cells and cytokine concentrations determined *via* Bio-plex array (Bio-Rad Laboratories) or ELISA (eBioscience). Analyses were performed following manufacturer's instructions and cytokine concentrations calculated using standard curves generated within the same assay. Antigen-specific T-cell IFNγ secretion/accumulation was assessed in splenocytes cultured in the presence of gp140 (16 μg/mL) over 3–5 days. Secreted IFNγ was detected by ELISA (eBioscience) and the frequencies of antigen specific IFNγ producing T-cells were detected by IFNγ cytokine secretion assay (Miltenyi Biotec).

### 2.4 *In vitro* stimulation

Murine RAW-Blue and human THP-1-CD14-Blue (Invivogen) reporter cells expressing NFκB/AP-1 transcription factor-induced embryonic alkaline phosphatase (SEAP) were used to detect PRR stimulation. Cells were cultured for 24 h in the presence of PBS, Carbopol or a variety of PRR ligands, supernatants harvested and co-incubated with SEAP detection reagent Quantiblue (Invivogen). Inflammasome activation was assessed after overnight incubation in the presence or absence of 25 ng/mL *Escherichia coli* LPS (Sigma–Aldrich, USA), followed by extensive washing to remove trace LPS prior to *in vitro* adjuvant stimulation (4 h, or (ATP) 30 min). Supernatants were frozen and cells lysed in RIPA buffer (Life Technologies) containing protease inhibitors. Lysates were directly loaded in Laemmli buffer and gels semi-dry transferred to nitrocellulose membranes (Amersham Biosciences, UK). Membranes were blocked with 5% milk in PBS (Oxoid, UK) and caspase-1 detected by polyclonal rabbit anti-caspase-1 antibody (Santa Cruz, USA) and anti-rabbit IgG-HRP (Serotec, USA).

Please refer to the supplementary materials for additional methods.

## 3 Results

### 3.1 Carbopol triggers immune cell recruitment and potently induces pro-inflammatory chemokines and cytokines

**Fig. 1:**
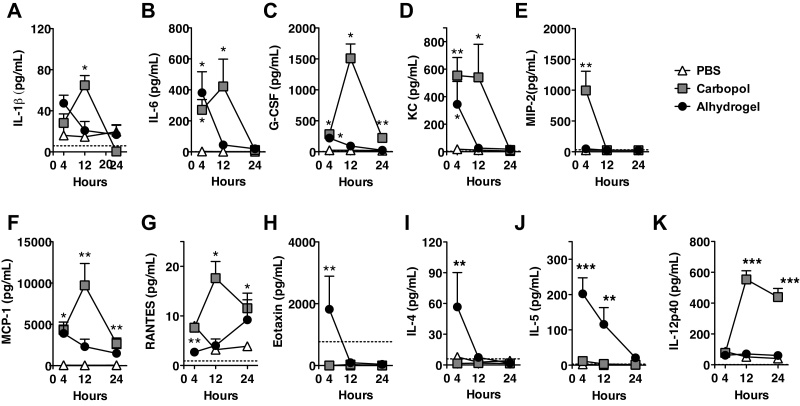
Carbopol induces pro-inflammatory chemokines *in vivo*. (A–K) Groups of 4–5 Balb/c mice were injected i.p. with PBS, Carbopol (1 mg) or Alhydrogel (1 mg Al^3+^) followed by a small volume peritoneal lavage at 4, 12 or 24 h. Cells were removed from lavage samples by centrifugation and peritoneal cytokine/chemokine titers quantified by multi-analyte profiling bead technology. Mean protein concentrations are shown ± SEM. Dashed lines (where visible) represent the limit of detection and significance was determined by two-way ANOVA against PBS controls with Bonferroni correction (*p < 0.05, **p < 0.01, ***p < 0.001).

To investigate the potential mode of action of Carbopol, we compared early innate immune responses induced by Carbopol with those induced by Alhydrogel, which elicits a strong Th2/IgG1-associated immune bias [24]. Since cellular recruitment is increasingly recognized as having a major influence on adjuvanticity and adaptive immune outcome in response to adjuvants [4], [6], [8], [10], [25], [26], [27], we assessed Carbopol-induced chemokine and cytokine secretion using the intraperitoneal (i.p.) administration model. Although this does not represent a human immunization route, it nevertheless represents a useful model for mechanistic analysis [8], [21]. Strong responses were detected in Carbopol recipients (Fig. 1), with significant induction of IL-1β, IL-6, G-CSF, KC, MIP-2, MCP-1 and RANTES (Fig. 1A–G). These chemokine trends were shared by Alhydrogel-adjuvanted mice, which also displayed significant but less potent induction of IL-6, G-CSF, KC, and MCP-1. One notable exception was the eosinophil recruitment factor Eotaxin, secreted at high levels by Alhydrogel-treated mice, but absent in Carbopol recipients (Fig. 1H). By contrast, the pattern of cytokine secretion associated with T-cell polarization was strikingly different between Carbopol and Alhydrogel groups (Fig. 1I–K). Alhydrogel recipients expressed significantly elevated levels of the Th2-associated cytokines IL-4 and IL-5, whereas Carbopol conversely elaborated significant quantities of the Th1-associated cytokine IL-12p40. Time course analyses revealed differing kinetics within the response to Alhydrogel and Carbopol, with chemokines/cytokine concentrations peaking at 4 h and 12 h respectively.

**Fig. 2:**
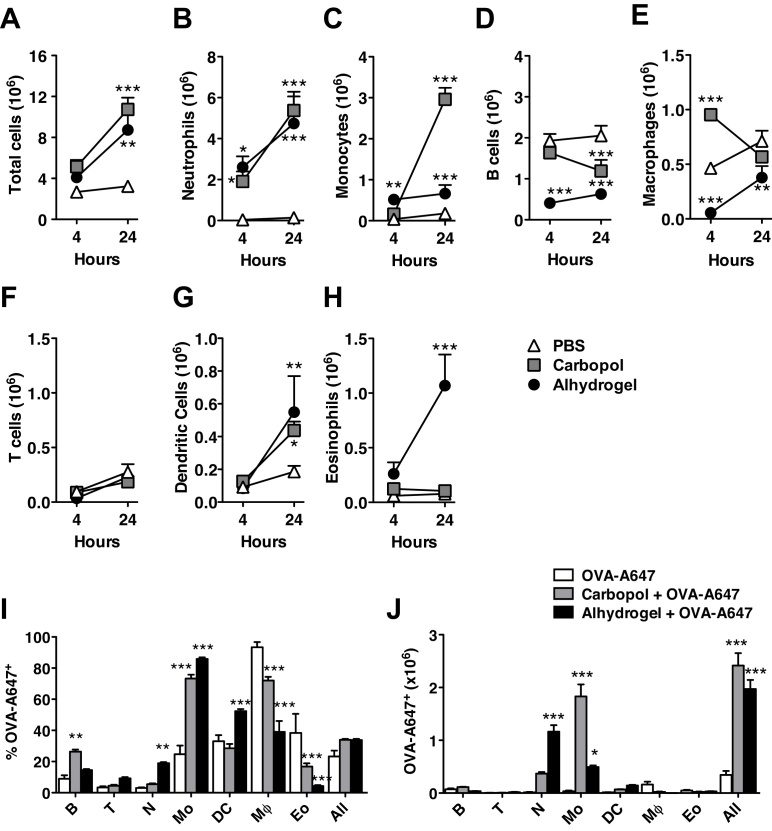
Carbopol induces inflammatory cell recruitment and antigen uptake at the site of immunization. (A–H) Groups of 4–5 Balb/c mice were injected i.p. with PBS, Carbopol (1 mg) or Alhydrogel (1 mg Al^3+^). Peritoneal lavages were performed after 4 and 24 h and leukocytes stained for flow cytometry to determine the absolute numbers of individual leukocyte populations. The mean absolute number of each population is shown ± SEM and significance tested by two-way ANOVA against PBS controls with Bonferroni correction. (I–J) Groups of 5 Balb/c mice were injected i.p. with PBS, Carbopol or Alhydrogel in the presence or absence of ovalbumin-Alexa-647 (OVA-A647) as indicated. After 24 h, peritoneal lavages were performed and leukocytes identified by flow cytometry. (I) The percentage of each population found associated with labeled antigen and (J) the absolute numbers of antigen positive cells are shown. Histogram bars represent the mean ± SEM and significance was tested by one-way ANOVA followed by Bonferroni correction (*p < 0.05, **p < 0.01, ***p < 0.001).

We next analyzed cellular recruitment post i.p. administration (Fig. 2A–H). Both Carbopol and Alhydrogel elicited a large influx of cells within 24 h, with significant increases in neutrophil, monocyte and DC numbers, but striking differences were observed between the two adjuvants. Of particular significance were the high numbers of monocytes but low numbers of eosinophils recruited into the peritoneum of Carbopol-injected mice (Fig. 2C and H), compared to the opposing phenotype in the Alhydrogel group. Alhydrogel recipients displayed a high degree of peritoneal eosinophilia and significantly lower numbers of monocytes compared to Carbopol recipients (*p* < 0.001). A decline in resident B cell and macrophage numbers in the peritoneum is often associated with cellular activation (28) and this can be seen after 4 h in Alhydrogel treated mice when compared to PBS controls (Fig. 2D–E). However, this effect was delayed in Carbopol-treated mice, in which mature macrophage numbers were transiently increased 4 h after Carbopol injection, prior to a significant reduction at 24 h when compared to PBS treated control mice. These data suggest that whilst both Carbopol and Alhydrogel drive significant leukocyte recruitment and inflammation early post-immunization, their mechanisms of action are likely to be distinct.

### 3.2 Carbopol promotes antigen uptake by APC

To investigate antigen uptake in the presence of Carbopol, mice were injected with fluorescently labeled ovalbumin (OVA) with or without adjuvant (Fig. 2I–J). 24 h later, antigen was detected in association with multiple cell types, but principally with monocytes, macrophages and DC (Fig. 2I). Absolute numbers of antigen-positive cells increased approximately five-fold with Carbopol or Alhydrogel (Fig. 2J). Despite similarities in the absolute number of antigen positive cells in the presence of either adjuvant, uptake was biased toward distinct leukocyte populations: Carbopol drove antigen uptake primarily in monocytes, whereas Alhydrogel predominantly drove neutrophil uptake. Whilst differences in monocyte-associated antigen uptake may reflect the increased presence of monocytes in the Carbopol-stimulated mice, the equal frequencies of neutrophils present in both groups suggest additional bias toward antigen uptake by neutrophils in Alhydrogel-stimulated mice. These data suggest there may be differing mechanisms of antigen uptake induced by these adjuvants.

### 3.3 Carbopol drives Th1 responses in the absence of TLR activation

**Fig. 3:**
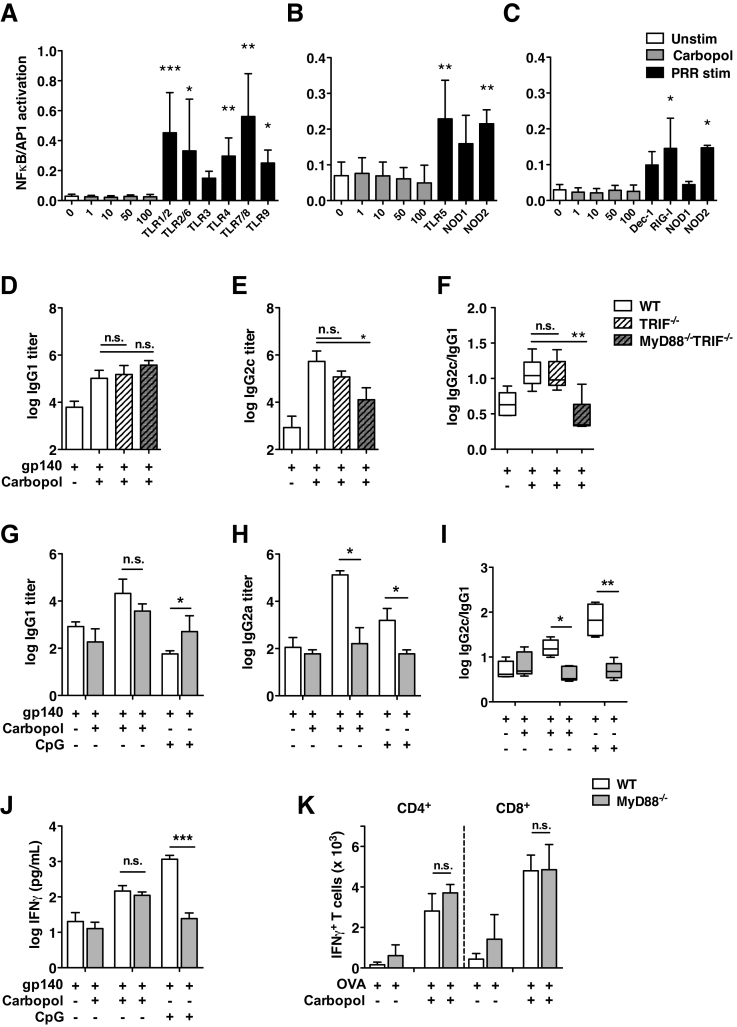
Carbopol adjuvanticity is independent of PRR signaling. NFκB/AP-1 activation was measured in (A and B) RAW-Blue macrophages or (C) THP-1-CD14-Blue monocytes in response to Carbopol or a range of PRR agonists ligands: TLR1/2 (Pam3CSK4, 100 ng/mL), TLR2 (LTA 100 ng/mL), TLR2/6 (FSL-1, 100 ng/mL), TLR3 (poly I:C 10 μg/mL), TLR4 (LPS, 1 μg/mL), TLR5 (Flagellin, 1 μg/mL), TLR7/8 (Imiquimod, 5 μg/mL), TLR9 (CpG, 1 μg/mL), NOD1 (Tri-DAP, 10 μg/mL), NOD2 (MDP, 10 μg/mL), Dectin-1 (Zymosan, 100 μg/mL) and RIG-I (CL075, 300 ng/mL). Histogram bars represent the mean NFκB/AP-1 activation observed after 24 h stimulation pooled from *n* ≥ 5 independent experiments ± SD. Significance was tested by Kruskal–Wallis one-way ANOVA. (D–F) Groups of 5 WT, TRIF^−/−^ and MyD88^−/−^TRIF^−/−^ mice were subcutaneously primed and boosted (week 4) with 2 μg gp140 alone or emulsified in Carbopol (1 mg). Serum endpoint titers of gp140-specific antibodies 2 weeks post-boost are shown for (D) IgG1, (E) IgG2c and (F) the ratio of log transformed IgG2a:IgG1 isotypes. (G–I) Groups of 5 WT and MyD88^−/−^ mice were primed subcutaneously (s.c.) and boosted (week 3) with 5 μg gp140 alone, or in the presence of Carbopol (1 mg) or CpG-ODN (30 μg). Serum endpoint titers of gp140-specific antibodies 2 weeks post-boost are shown for (G) IgG1, (H) IgG2a and (I) the ratio of log-transformed IgG2c:IgG1 isotypes. Bars represent mean endpoint titers ± SD, box and whiskers represent the median ± minimum to maximum values. Significance was determined by one-way ANOVA with Bonferroni or Kruskal–Wallis analyses as described in materials and methods. (J–K) Groups of 4–5 WT or MyD88^−/−^ mice were immunized s.c. with either (J) 5 μg gp140 alone or with Carbopol (0.5 mg) or CpG-ODN (30 μg), or (K) 2 μg OVA alone or with Carbopol. Six weeks after immunization, splenocytes were cultured with either (J) 16 μg/mL gp140 or (K) 20 μg/mL OVA for 5 days and IFNγ secretion measured by (J) cytokine ELISA or (K) flow cytometry. Histogram bars represent the mean ± SD. Significance was determined by one-way ANOVA with Bonferroni correction (*p < 0.05, **p < 0.01, ***p < 0.001, n.s. *p* > 0.05).

Given that Th1-biased adaptive immunity is commonly linked with adjuvants that drive pattern recognition receptor (PRR), in particular TLR [28], [29] activation, we investigated whether Carbopol might drive inflammation *via* this route using monocyte/macrophage NFκB/AP-1 reporter cell lines expressing multiple PRRs. Whilst these cells responded to a variety of PRR ligands, there was no response to overnight culture with increasing doses of Carbopol (Fig. 3A–C).

To confirm this lack of PRR activation *in vivo*, mice deficient in expression of the TLR downstream signaling molecules MyD88 and TRIF were immunized subcutaneously (s.c.) with HIV-1 gp140 alone or with Carbopol. Antigen-specific antibody responses in TRIF^−/−^ mice were unchanged, demonstrating that Carbopol adjuvant activity does not require TRIF-dependent TLR activation (Fig. 3D–F). By contrast, a significant reduction in antigen-specific IgG2c titers was observed in MyD88^−/−^TRIF^−/−^ double-deficient mice immunized in the presence of Carbopol, resulting in a significant shift toward an IgG1 isotype bias. Since we had excluded a requirement for TRIF signaling, we assessed antibody responses to Carbopol in MyD88^−/−^ mice in comparison with the TLR9 ligand CpG and observed a similarly weak IgG2a response and IgG1 isotype bias (Fig. 3G–I). It has been reported that MyD88 is also involved in non-PRR signaling pathways including stabilization of IFNγ receptor signaling [39] and B-cell immunoglobulin class switching [30]. We therefore directly assessed Carbopol-driven T-cell polarization in the absence of MyD88. To this end, IFNγ secretion (Fig. 3J) and the frequency of antigen-specific IFNγ-secreting CD4^+^ T-cells (Fig. 3K) were quantified in gp140-stimulated splenocytes isolated from immunized mice. Strikingly, unlike the TLR9 agonist CpG, Th1 polarization in response to Carbopol-adjuvanted immunization was intact in the absence of MyD88. Therefore, reduced IgG2a-biased humoral responses to Carbopol in the absence of MyD88 are not due to defective development of antigen-specific Th1 cells, but probably result from MyD88-dependent TLR-independent signaling pathways.

### 3.4 Carbopol activates the inflammasome but its adjuvant activity is independent of NLRP3 and Caspase 1

**Fig. 4:**
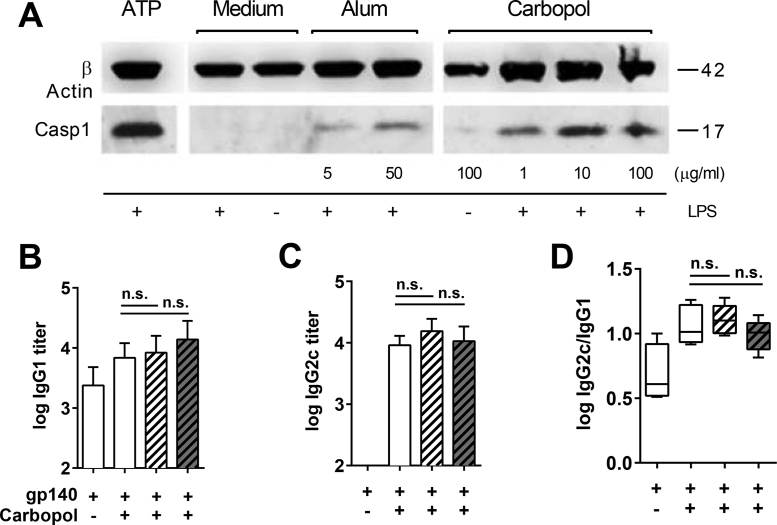
Carbopol adjuvant activity is independent of inflammasome activation. (A) Unstimulated or pre-stimulated (LPS 25 ng/mL) BMDM were incubated for 4 h with increasing doses of Carbopol or Alhydrogel as indicated and mature Caspase-1 detected by Western blot. (B–D) Groups of 5 WT (open bars), NLRP3^−/−^ (hatched bars) and Caspase1^−/−^ (dark hatched bars) mice were primed and boosted s.c. (week 3) with 2 μg gp140 alone or mixed with Carbopol (1 mg). Serum endpoint titers of gp140-specific antibodies 3 weeks post-boost are shown for (B) IgG1, (C) IgG2c and (D) the ratio of log-transformed IgG2a:IgG1 isotypes. Bars represent mean endpoint titers ± SD, box and whiskers represent the median ± minimum to maximum values. Significance was determined by one-way ANOVA with Bonferroni correction (n.s. *p* > 0.05).

Inflammasome activation contributes to the adjuvant activity of Alhydrogel [26], [31], [32], [33]. Since we observed significant increases in IL-1β secretion in response to Carbopol, which is a signature cytokine for inflammasome activation [4], [8], [34], we investigated Carbopol-induced Caspase 1 maturation *in vitro*. Mature, cleaved Caspase-1 was detected in BMDM stimulated with either Carbopol or Alhydrogel, demonstrating dose-dependent inflammasome activation (Fig. 4A). However, no significant differences were observed in antigen-specific IgG1 and IgG2a titers between WT, inflammasome-deficient NLRP3^−/−^ and Caspase-1^−/−^ mice (Fig. 4B–D). These data suggest that although Carbopol is a potent inflammasome activator, this pathway lacks a central role in driving antigen-specific adaptive immunity in response to Carbopol.

### 3.5 Carbopol is captured and phagocytosed by antigen presenting cells

**Fig. 5:**
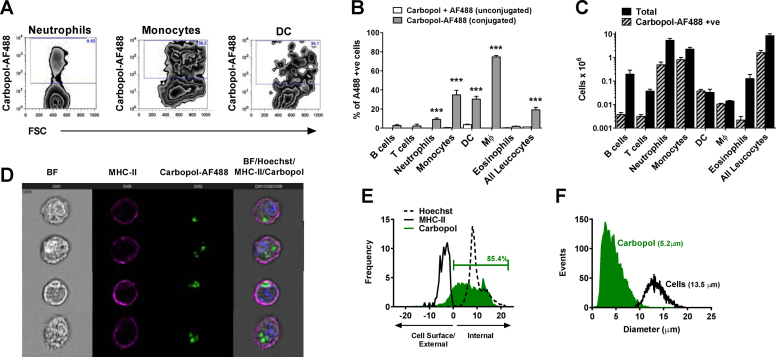
Carbopol is captured by antigen presenting cells *in vivo* and *in vitro*. (A–C) Groups of 4–5 Balb/c mice were injected i.p. with either unconjugated Carbopol (1 mg) and free fluorescent label (Carbopol + AF488, control) or Carbopol covalently conjugated to fluorescent-label (Carbopol–AF488). After 24 h, peritoneal lavages were performed and leukocytes identified by flow cytometry. (A) Representative density plots showing *in vivo* fluorescent Carbopol uptake by phagocytes. (B) Percentage of each leukocyte population associated with labeled adjuvant and (C) the absolute numbers of adjuvant positive cells. Histogram bars represent the mean ± SEM and significance was tested by one-way ANOVA followed by Bonferroni correction (*p < 0.05, **p < 0.01, ***p < 0.001). (D–F) BMDC were cultured for 16 h in the presence of fluorescently labeled Carbopol before surface (MHC-II) and nuclear (Hoechst) staining and Imagestream analysis. (D) Representative gallery of Carbopol positive cells determined by co-localization with an intracellular mask as described in materials and methods. Histogram representation of (E) the frequency of BMDC with intracellular Carbopol–AF488 and (F) the mean and distribution of individual cell and Carbopol–AF488 particle diameters as measured by Imagestream.

Since charged particles are preferentially taken up by phagocytes including antigen presenting cells, we investigated interactions between leukocytes and Carbopol *in vivo* and *in vitro* (Fig. 5) *via* covalently-conjugated fluorescent Carbopol (Carbopol–AF488). To control for any potential increase in cellular auto-fluorescence or uptake of residual unbound label (AF488), an unconjugated formulation of Carbopol was prepared containing equivalent quantities of Carbopol and fluorescent label (Carbopol + AF488). We observed significant increases in Carbopol–AF488 positive cells within the major phagocytic leukocyte populations of neutrophils, monocytes, macrophages and DC (Fig. 5A–C) after i.p. administration. To establish whether Carbopol–cell interactions represented Carbopol surface binding or internalization, we co-cultured BMDC with Carbopol–AF488 *in vitro* and utilized high-throughput imaging (Imagestream) (Fig. 5D). These analyses demonstrated that in >85% of Carbopol–AF488 positive cells the adjuvant was localized intracellularly and that the mean Carbopol particle diameter was 5.2 μm after phagocytosis (Fig. 5E–F). In many cases, individual cells had phagocytosed multiple Carbopol particles, resulting in significant proportions of the intracellular space being occupied by the adjuvant.

### 3.6 Internalization into the phagolysosome promotes conformational change in Carbopol and reactive oxygen species production

**Fig. 6:**
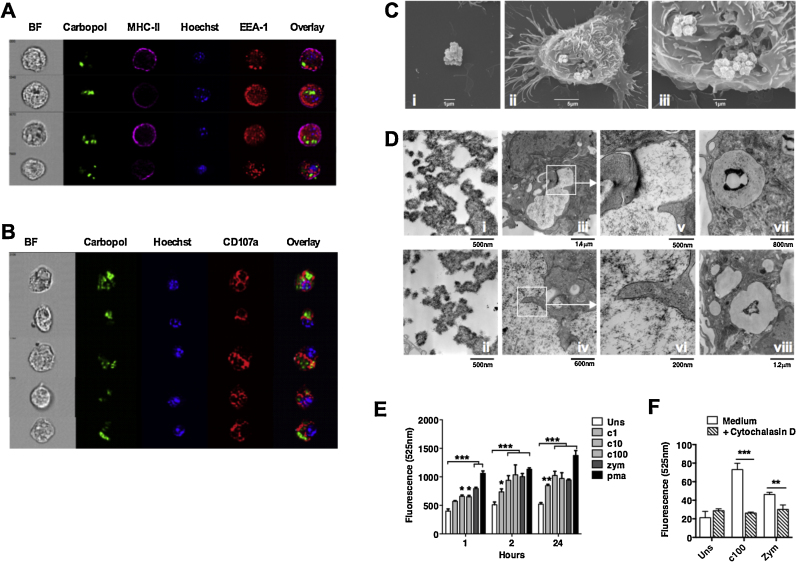
Carbopol drives ROS production in APC in a phagocytosis dependent manner. (A and B) BMDC were cultured for 16 h in the presence of fluorescently-labeled Carbopol followed by surface staining (MHC-II) and intracellular staining (Hoechst/EEA1/CD107a) prior to Imagestream analysis. Representative galleries of Carbopol positive cells co-stained with (A) EEA1 or (B) CD107a are shown. (C and D) BMDC were cultured for 16 h in the presence of either Carbopol or TLR ligand-depleted Zymosan and imaged by electron microscopy. (C) Representative SEM images of (i) extracellular Carbopol and (ii and iii) the early stages of Carbopol phagocytosis. (D) Representative TEM images of (i and ii) extracellular Carbopol, (iii and vi) phagocytosed Carbopol and (vii and viii) phagocytosed Zymosan. (E and F) ROS detection was performed using Carboxy-H2DCFDA labeling followed by spectrophotometer analysis at 525 nm. (E) ROS production was assessed in BMDM cultured for 1, 2, or 24 h in the presence of either 1–100 μg/mL Carbopol, 10 μg/mL Zymosan or 10 μM PMA. (F) ROS production was assessed in BMDC cultured for 4 h with either 100 μg/mL Carbopol or 10 μg/mL Zymosan in the presence or absence of Cytochalasin D (10 μM).

Given the minimally-biodegradable nature of Carbomers, we examined the effects of Carbopol phagocytosis and the ensuing inflammatory response. Using high-throughput imaging we assessed subcellular localization of Carbopol after phagocytic uptake by BMDC (Fig. 6A and B). Carbopol particles did not co-localize with the early-endosomal marker EEA1 [35], but were predominantly located within CD107a^+^ lysosomal compartments [36]. Using electron microscopy we observed phagocytic cup formation and subsequent localization of Carbopol within compartments with lysosomal morphology (Fig. 6C and D). Interestingly, we observed substantial morphological modifications indicative of conformational change in Carbopol within the lysosomal compartment. Whereas extracellular Carbopol formed a very tightly packed ‘closed’ structure, lysosomal material adopted an ‘open’ mesh-like conformation (Fig. 6Di-ii, iii-vi). It has been previously established that lysosomal accumulation of non-biodegradable particles and/or lysosomal disruption can drive cellular activation and inflammation, and a key signature of this response is ROS production [32], [37]. We therefore compared Carbopol-induced ROS production in BMDC and BMDM *in vitro* with well-characterized particulate and non-particulate controls (Fig. 6E and F). We detected significant BMDM-derived ROS production in response to escalating concentrations of Carbopol, which increased in a dose- and time-dependent manner (Fig. 6E). Significant ROS production was also induced by Carbopol exposure to BMDC, and this response was completely abrogated by the presence of the actin polymerization inhibitor Cytochalasin D, which prevented Carbopol uptake (Fig. 6F). Taken together, these data suggest that phagocytosis results in Carbopol accumulation and conformational modification within lysosomal compartments, triggering ROS production and inflammation.

## 4 Discussion

The successful development of vaccines against pathogens refractory to current vaccination strategies such as HIV-1, HCV and Malaria will be partly dependent upon new adjuvant technologies [38]. Although Carbopol has been used in veterinary vaccines for many years, the cellular mechanisms that drive its efficacy in adjuvant applications are yet to be characterized and therefore investigation of the mode of action of Carbopol is warranted, particularly if this adjuvant is to be taken into man.

We demonstrate that Carbopol adjuvant activity is accompanied by potent innate immune activation, comprising chemokine and pro-inflammatory cytokine secretion associated with rapid leukocyte recruitment and antigen uptake. Striking differences were observed in early immune responses to Carbopol and Alhydrogel, consistent with the adaptive immune bias reported for these adjuvants [3], [22]. Similar contrasting responses have been reported between TLR and non-TLR adjuvants [39], suggesting that the innate response to Carbopol more closely resembles that of a TLR-targeted formulation. However, both *in vitro* and *in vivo* analyses demonstrated that Carbopol does not directly trigger TLR activation and the downstream signaling pathways associated with pathogen recognition. In addition, Th1 induction was intact in the absence of TLR-Myd88/TRIF signaling, demonstrating a non-canonical pathway of Carbopol-induced Th1 polarization. Whilst the TLR-Myd88/TRIF signaling axis is the best characterized mechanism of Th1 induction, other pathways of APC activation and resultant Th1-polarization have also been reported, particularly in response to infection [7], [40], [41], [42]. Although the alternate receptors and signaling pathways involved remain to be fully explored, some non-TLR receptors such as C-type lectins (*e.g.* Mincle, MCL, Mannose receptor) and scavenger receptors can drive Th1 responses *via* both Myd88-dependent and -independent signaling [43], [44], [45], [46]. Given the polyanionic structure of carbomers it is possible that Carbopol is recognized *via* one such pathway, which will require further investigation. Carbopol and Alhydrogel yield strikingly different Th biases, with Carbopol and Alhydrogel eliciting robust Th1 and Th2 responses respectively. We demonstrate that these adaptive immune responses are mirrored by the early innate chemokine and cytokine responses that predict the adaptive immune outcome. Thus Carbopol selectively triggers MIP-2 MCP-1, RANTES and IL-12p40 release, whereas Alhydrogel drives rapid (within 4 h) Eotaxin, IL-4 and IL-5 release.

As with other immune adjuvants, Carbopol efficiently induced inflammasome formation. However since Carbopol-induced humoral responses were independent of this process, we conclude that inflammasome activation is secondary to other Carbopol-triggered mechanisms of immune activation. Since it has been reported that phagolysosomal destabilization after adjuvant phagocytosis is an important step in inflammasome activation [32], we investigated the interactions between Carbopol and phagocytes in more detail. Carbopol was avidly captured by a range of phagocytes including APC, most likely due to its particulate nature and strong negative charge which may be selectively recognized by scavenger and other receptors on phagocytes [47]. Uptake was accompanied by a conformational change in Carbopol structure similar to that described for a range of homopolymers at low pH, including carbomers, which make these compounds suitable for controlled-release drug delivery [48]. Due to the polyanionic nature of Carbopol, a reduction in pH causes the carboxylate moieties on the polymer backbone to lose charge, eliminating the repulsive electrostatic forces and collapsing the gel matrix. It is likely that the progressive acidification of the lysosome occurring during lysosomal maturation [49] drives the Carbopol conformational change. In addition, interference with lysosomal acidification has been previously reported in response to phagocytic uptake of polyanionic compounds [50]. In line with this, we found a clear correlation between Carbopol phagocytosis and ROS production by APC, which is a hallmark of lysosomal arrest and disruption. We therefore conclude that phagocytosis of the adjuvant plays an early and critical role in the ensuing inflammatory response to Carbopol administration.

The Th1 polarized response induced by Carbopol in the absence of TLR stimulation is uncommon and warrants further investigation to understand the molecular mechanisms at play. Further work on this type of adjuvant will elucidate mechanisms and lead the way to the design of adjuvants with increased potency and reduced toxicity.

## References

[1] Pulendran B., Ahmed R. (2006). Translating innate immunity into immunological memory: implications for vaccine development. Cell.

[2] Pashine A., Valiante N.M., Ulmer J.B. (2005). Targeting the innate immune response with improved vaccine adjuvants. Nat Med.

[3] Eisenbarth S.C., Colegio O.R., O’Connor W., Sutterwala F.S., Flavell R.A. (2008). Crucial role for the Nalp3 inflammasome in the immunostimulatory properties of aluminium adjuvants. Nature.

[4] De Gregorio E., D’Oro U., Wack A. (2009). Immunology of TLR-independent vaccine adjuvants. Curr Opin Immunol.

[5] Marichal T., Ohata K., Bedoret D., Mesnil C., Sabatel C., Kobiyama K. (2011). DNA released from dying host cells mediates aluminum adjuvant activity. Nat Med.

[6] Wegmann F., Gartlan K.H., Harandi A.M., Brinckmann S.A., Coccia M., Hillson W.R. (2012). Polyethyleneimine is a potent mucosal adjuvant for viral glycoprotein antigens. Nat Biotechnol.

[7] Gavin A.L., Hoebe K., Duong B., Ota T., Martin C., Beutler B. (2006). Adjuvant-enhanced antibody responses in the absence of toll-like receptor signaling. Science.

[8] Lambrecht B.N., Kool M., Willart M.A., Hammad H. (2009). Mechanism of action of clinically approved adjuvants. Curr Opin Immunol.

[9] Uto T., Wang X., Sato K., Haraguchi M., Akagi T., Akashi M. (2007). Targeting of antigen to dendritic cells with poly(gamma-glutamic acid) nanoparticles induces antigen-specific humoral and cellular immunity. J Immunol.

[10] Mosca F., Tritto E., Muzzi A., Monaci E., Bagnoli F., Iavarone C. (2008). Molecular and cellular signatures of human vaccine adjuvants. Proc Natl Acad Sci U S A.

[11] Diamantstein T., Wagner B., Beyse I., Odenwald M.V., Schulz G. (1971). Stimulation of humoral antibody formation by polyanions: I. The effect of polyacrylic acid on the primary immune response in mice immunized with sheep red blood cells. Eur J Immunol.

[12] Gall D., Knight P.A., Hampson F. (1972). Adjuvant activity of polyelectrolytes. Immunology.

[13] Gualandi G.L., Losio N.M., Muratori G., Foni E. (1988). The ability by different preparations of porcine parvovirus to enhance humoral immunity in swine and guinea pigs. Microbiologica.

[14] Hoogland M.J., Opriessnig T., Halbur P.G. (2006). Effects of adjuvants on porcine circovirus type 2-associated lesions. J Swine Health Prod.

[15] Liu I.K., Turner J.W., Van Leeuwen E.M., Flanagan D.R., Hedrick J.L., Murata K. (2005). Persistence of anti-zonae pellucidae antibodies following a single inoculation of porcine zonae pellucidae in the domestic equine. Reproduction.

[16] Mair K.H., Koinig H., Gerner W., Hohne A., Bretthauer J., Kroll J.J. (2015). Carbopol improves the early cellular immune responses induced by the modified-life vaccine Ingelvac PRRS((R)) MLV. Vet Microbiol.

[17] Mumford J.A., Wilson H., Hannant D., Jessett D.M. (1994). Antigenicity and immunogenicity of equine influenza vaccines containing a Carbomer adjuvant. Epidemiol Infect.

[18] Tollersrud T., Norstebo P.E., Engvik J.P., Andersen S.R., Reitan L.J., Lund A. (2002). Antibody responses in sheep vaccinated against *Staphylococcus aureus* mastitis: a comparison of two experimental vaccines containing different adjuvants. Vet Res Commun.

[19] Lai R.P., Seaman M.S., Tonks P., Wegmann F., Seilly D.J., Frost S.D. (2012). Mixed adjuvant formulations reveal a new combination that elicit antibody response comparable to Freund's adjuvants. PLoS ONE.

[20] Dey A.K., Burke B., Sun Y., Hartog K., Heeney J.L., Montefiori D. (2012). Use of a polyanionic carbomer, Carbopol971P, in combination with MF59, improves antibody responses to HIV-1 envelope glycoprotein. Vaccine.

[21] Wegmann F., Moghaddam A.E., Schiffner T., Gartlan K.H., Powell T.J., Russell R.A. (2015). The Carbomer–Lecithin adjuvant adjuplex has potent immunoactivating properties and elicits protective adaptive immunity against influenza virus challenge in mice. Clin Vaccine Immunol.

[22] Krashias G., Simon A.K., Wegmann F., Kok W.L., Ho L.P., Stevens D. (2010). Potent adaptive immune responses induced against HIV-1 gp140 and influenza virus HA by a polyanionic carbomer. Vaccine.

[23] Bowles E.J., Schiffner T., Rosario M., Needham G.A., Ramaswamy M., McGouran J. (2014). Comparison of neutralizing antibody responses elicited from highly diverse polyvalent heterotrimeric HIV-1 gp140 cocktail immunogens versus a monovalent counterpart in rhesus macaques. PLoS ONE.

[24] Oleszycka E., Lavelle E.C. (2014). Immunomodulatory properties of the vaccine adjuvant alum. Curr Opin Immunol.

[25] Calabro S., Tortoli M., Baudner B.C., Pacitto A., Cortese M., O’Hagan D.T. (2011). Vaccine adjuvants alum and MF59 induce rapid recruitment of neutrophils and monocytes that participate in antigen transport to draining lymph nodes. Vaccine.

[26] Kool M., Soullie T., van Nimwegen M., Willart M.A., Muskens F., Jung S. (2008). Alum adjuvant boosts adaptive immunity by inducing uric acid and activating inflammatory dendritic cells. J Exp Med.

[27] Seubert A., Calabro S., Santini L., Galli B., Genovese A., Valentini S. (2011). Adjuvanticity of the oil-in-water emulsion MF59 is independent of Nlrp3 inflammasome but requires the adaptor protein MyD88. Proc Natl Acad Sci U S A.

[28] Barton G.M., Medzhitov R. (2002). Control of adaptive immune responses by Toll-like receptors. Curr Opin Immunol.

[29] Pulendran B. (2004). Modulating vaccine responses with dendritic cells and Toll-like receptors. Immunol Rev.

[30] He B., Santamaria R., Xu W., Cols M., Chen K., Puga I. (2010). The transmembrane activator TACI triggers immunoglobulin class switching by activating B cells through the adaptor MyD88. Nat Immunol.

[31] Eisenbarth S.C., Flavell R.A. (2009). Innate instruction of adaptive immunity revisited: the inflammasome. EMBO Mol Med.

[32] Hornung V., Bauernfeind F., Halle A., Samstad E.O., Kono H., Rock K.L. (2008). Silica crystals and aluminum salts activate the NALP3 inflammasome through phagosomal destabilization. Nat Immunol.

[33] Li H., Willingham S.B., Ting J.P., Re F. (2008). Cutting edge: inflammasome activation by alum and alum's adjuvant effect are mediated by NLRP3. J Immunol.

[34] Martinon F., Mayor A., Tschopp J. (2009). The inflammasomes: guardians of the body. Annu Rev Immunol.

[35] Mills I.G., Jones A.T., Clague M.J. (1999). Regulation of endosome fusion. Mol Membr Biol.

[36] Huynh K.K., Eskelinen E.L., Scott C.C., Malevanets A., Saftig P., Grinstein S. (2007). LAMP proteins are required for fusion of lysosomes with phagosomes. EMBO J.

[37] Dostert C., Petrilli V., Van Bruggen R., Steele C., Mossman B.T., Tschopp J. (2008). Innate immune activation through Nalp3 inflammasome sensing of asbestos and silica. Science.

[38] Rappuoli R. (2007). Bridging the knowledge gaps in vaccine design. Nat Biotechnol.

[39] Korsholm K.S., Petersen R.V., Agger E.M., Andersen P. (2009). T-helper 1 and T-helper 2 adjuvants induce distinct differences in the magnitude, quality and kinetics of the early inflammatory response at the site of injection. Immunology.

[40] Sukhumavasi W., Egan C.E., Warren A.L., Taylor G.A., Fox B.A., Bzik D.J. (2008). TLR adaptor MyD88 is essential for pathogen control during oral *Toxoplasma gondii* infection but not adaptive immunity induced by a vaccine strain of the parasite. J Immunol.

[41] Rivera A., Ro G., Van Epps H.L., Simpson T., Leiner I., Sant’Angelo D.B. (2006). Innate immune activation and CD4+ T cell priming during respiratory fungal infection. Immunity.

[42] Kursar M., Mittrucker H.W., Koch M., Kohler A., Herma M., Kaufmann S.H. (2004). Protective T cell response against intracellular pathogens in the absence of Toll-like receptor signaling via myeloid differentiation factor 88. Int Immunol.

[43] Singh S.K., Streng-Ouwehand I., Litjens M., Kalay H., Burgdorf S., Saeland E. (2011). Design of neo-glycoconjugates that target the mannose receptor and enhance TLR-independent cross-presentation and Th1 polarization. Eur J Immunol.

[44] Bhatia S., Mukhopadhyay S., Jarman E., Hall G., George A., Basu S.K. (2002). Scavenger receptor-specific allergen delivery elicits IFN-gamma-dominated immunity and directs established TH2-dominated responses to a nonallergic phenotype. J Allergy Clin Immunol.

[45] Richardson M.B., Williams S.J., Mincle M.C.L. (2014). C-type lectin receptors that sense damaged self and pathogen-associated molecular patterns. Front Immunol.

[46] Desel C., Werninghaus K., Ritter M., Jozefowski K., Wenzel J., Russkamp N. (2013). The Mincle-activating adjuvant TDB induces MyD88-dependent Th1 and Th17 responses through IL-1R signaling. PLoS ONE.

[47] Canton J., Neculai D., Grinstein S. (2013). Scavenger receptors in homeostasis and immunity. Nat Rev Immunol.

[48] (2014). Advanced polymers in medicine.

[49] Mindell J.A. (2012). Lysosomal acidification mechanisms. Annu Rev Physiol.

[50] Kielian M.C., Cohn Z.A. (1982). Intralysosomal accumulation of polyanions: II. Polyanion internalization and its influence on lysosomal pH and membrane fluidity. J Cell Biol.

