# Supplementary material for: Sterile inflammation induced by Carbopol elicits robust adaptive immune responses in the absence of pathogen-associated molecular patterns
Source: Vaccine. 2016 Apr 27;34(19):2188–96. doi: 10.1016/j.vaccine.2016.03.025 (PMC4850248; doi:10.1016/j.vaccine.2016.03.025)
Supplement: Supplementary file 1 [file mmc1.docx]

**SUPPLEMENTARY MATERIALS AND METHODS**

**Preparation of BMDC and BMDM**

Bone marrow derived macrophages (BMDM) were generated in culture supplemented with 15% L929-cell conditioned medium (LCM15) and fed at days 3 and 6 with fresh LCM15. BMDMs were harvested on day 7 by lifting with 4 mM Lidocaine (Sigma-Aldrich, USA) and macrophage purity determined by flow cytometry, with >90% cells staining F4/80^hi^CD11b^+^CD11c^-^MHC-II^int^. Bone marrow derived dendritic cells (BMDC) were generated in culture supplemented with 20 ng/mL recombinant murine GM-CSF (R&D Systems, USA). Cultures were fed at days 3 and 6 with fresh GM-CSF supplemented CM and harvested on day 7 by EDTA treatment.

**Imagestream Analysis**

BMDC were cultured for 16 h in the presence of either 100 µg/mL Carbopol or TLR ligand-depleted Zymosan (100 µg/mL) then washed and lifted prior to surface staining and fixation/permablisation (BD Biosciences). Cells were washed and stained with either Hoechst (0.5µg/mL, Molecular Probes), anti-EEA1 (rPolyclonal, Abcam) or biotinylated anti-CD107a (1D4B, Biolegend) as per manufacturer’s instructions. Secondary staining was performed with either streptavidin-TexasRed or anti-rabbit-Ig-TexasRed as required. Data was acquired by high throughput imaging on an Amnis Imagestream^X^-EDF. All acquisition was performed at 60 x magnification on low speed throughput. Ideas Software version 5.0 was employed for all offline analysis. For sub-cellular localization detection a mask was created that encompassed the cell but excluded the membrane and co-localization between this mask and the Carbopol signal was calculated. Carbopol particle and cell size were calculated using the ‘diameter’ feature using masking as appropriate.

**Electron microscopy**

BMDC were cultured on glass cover slips at 1-2 x 10^6^ cells/well for 4-16 h in the presence of either 100 µg/mL Carbopol or TLR ligand-depleted Zymosan (100 µg/mL). Cells were washed and then fixed with 2.5% glutaraldyehyde-100 mM cacodylate buffer prior to imaging. For scanning electron microscopy (SEM), samples were dehydrated through a series of ethanols and critically point-dried. After gold sputter coating, cells were examined in a JOEL JSM 6390 scanning electron microscope. For transmission electron microscopy (TEM), samples were negatively stained with 2% (wt/vol) uranyl acetate, dehydrated with ethanol and cells released from the plastic using propylene oxide. The cells were washed several times with propylene oxide and embedded in resin. Ultrathin (~70nm thick) sections were cut, stained with uranyl acetate and lead citrate then examined in a FEI Tecnai 12 electron microscope.

**Reactive Oxygen Species assay**

ROS production in BMDMs was determined using carboxy-H2DCFDA (Invitrogen). 1.5 x 10^6^ cells/well were cultured (1, 2 and 24 h) in phenol-red free medium either alone or in the presence of 100 µg/ml Carbopol, PMA (2 µM) or TLR ligand-depleted Zymosan (100 µg/mL). Following incubation, cells were washed with PBS and stained with carboxy-H2DCFDA (20 µM) in PBS for 10 mins at 37°C. Cells were washed again and lifted with EDTA (10µM)/Lidocaine (12 µM, 10 mins, 37°C), before resuspension in phenol-red free medium and transferred to 96 well black/clear bottom polystyrene cell culture microplates. Fluorescence intensity was measured with an excitation wavelength of 488 nm and an emission wavelength of 515 nm.

**Statistical analysis**

Statistical analyses were performed using Prism software (GraphPad, USA). Comparison of two sample data sets were carried out using the two-tailed Mann-Whitney test unless normal distribution was established, in which case an unpaired *t*-test was applied. Comparison of more than two data sets was carried out by one-way ANOVA using either Kruskal-Wallis or, if normal distribution was established, Bonferroni post-tests for inter-group analysis. Abnormally distributed serum immunoglobulin titers were log transformed prior to one-way ANOVA analysis with post-tests as described above. The statistical test used for each individual experiment and associated *p* values are indicated in corresponding figure legends.
